# SOCS2 overexpression alleviates diabetic nephropathy in rats by inhibiting the TLR4/NF-κB pathway

**DOI:** 10.18632/oncotarget.20434

**Published:** 2017-08-24

**Authors:** Suxia Yang, Junwei Zhang, Shiying Wang, Xinxin Zhao, Jun Shi

**Affiliations:** ^1^ Department of Nephrology, Huaihe Hospital of Henan University, Kaifeng, 475000, China

**Keywords:** diabetic nephropathy, SOCS2, renal injury, TLR4/NF-Tβ pathway, inflammatory cytokines

## Abstract

Suppressor of cytokine signaling 2 (SOCS2) was reported to be involved in the development of Diabetic Nephropathy (DN). However, its underlying mechanism remains undefined. Western blot was carried out to determine the expressions of SOCS2, Toll-like receptors 4 (TLR4) and nuclear factor kappa B (NF-κB) pathway-related proteins in DN patients, streptozotocin (STZ)-induced DN rats and high glucose (HG)-stimulated podocytes. The effects of SOCS2 overexpression on renal injury, the inflammatory cytokines production, renal pathological changes, apoptosis and the TLR4/NF-κB pathway in DN rats or HG-stimulated podocytes were investigated. TLR4 antagonist TAK-242 and NF-κB inhibitor PDTC were used to confirm the functional mechanism of SOCS2 overexpression in HG-stimulated podocytes. SOCS2 was down-regulated, while TLR4 and NF-κB were up-regulated in renal tissues of DN patients and DN rats. Ad-SOCS2 infection alleviated STZ-induced renal injury and pathological changes and inhibited STZ-induced IL-6, IL-1β and MCP-1 generation and activation of the TLR4/NF-κB pathway in DN rats. SOCS2 overexpression attenuated apoptosis, suppressed the inflammatory cytokines expression, and inactivated the TLR4/NF-κB pathway in HG-stimulated podocytes. Suppression of the TLR4/NF-κB pathway enhanced the inhibitory effect of SOCS2 overexpression on apoptosis and inflammatory cytokines expressions in HG-stimulated podocytes. SOCS2 overexpression alleviated the development of DN by inhibiting the TLR4/NF-κB pathway, contributing to developing new therapeutic strategies against DN.

## INTRODUCTION

Diabetic nephropathy (DN) is a complicated diabetic disease that can develop into a progressive fibrosing kidney disease [1]. Despite improved prognosis in the last few years, DN has become a leading cause of end-stage renal disease (ESRD) and even mortality in the industrialized world, and its escalating incidence and prevalence in parallel with the pandemic of type 2 diabetes are great threats to life [2, 3]. Many studies suggest that DN patients are usually accompanied with some pathological signs, such as thicker glomerular basement membrane, renal hypertrophy, progressive glomerulosclerosis and mesangial expansion [4]. Although DN is historically regarded as a largely nonimmune disease, evidence from recent experimental and clinical studies indicates that chronic inflammation exerts a crucial role in DN pathogenesis and progression [5]. It is stated that DN has been related to multiple pathological factors such as glucose metabolic disorder, abnormal expression of cytokines, inflammatory factors and oxidative stress [6]. Although the treatment for DN is important to improve patients’ prognosis, further researches concerning the potential mechanism of intrarenal inflammation in aggravating DN are essential to be investigated, which may provide novel therapeutic targets for anti-infammatory strategies against DN.

Suppressor of cytokine signaling (SOCS) is implicated in the negative regulation of the signal transduction of cytokines through suppressing the Janus kinase (JAK)/signal transducers and activators of transcription (STAT) signaling pathway. It is well documented that SOCS1 and SOCS3 play a protective role in the development of DN by suppressing the JAK/STAT signaling pathway [7]. As another member of SOCS family, SOCS2 was initially characterized for its role in the periphery as a negative regulator of growth hormone (GH) signaling [8]. SOCS2 was reported to inhibit the activation of the JAK/STAT signaling pathway and reduce the expression of inflammatory cytokines and fibrosis associated proteins in DN [9]. However, its underlying mechanism remains unclear.

Toll-like receptors (TLRs) function as important receptors of the innate immune system through recognizing highly conserved microbial molecules known as pathogen associated molecular patterns (PAMPs) on microorganisms [10]. TLRs exert a fundamental role in the innate immune system via activating proinflammatory pathways in response to microbial pathogens [11]. TLRs activated by exogenous pathogens released by damaged or stressed tissues such as heat-shock proteins (HSPs) improve the activity of nuclear factor kappa B (NF-κB) and increase the release of pro-inflammatory cytokines and chemokines, thus activating downstream inflammation cascades and initiating the adaptive immune response [12, 13]. These up-regulated proinflammatory cytokines and chemokines, such as interleukin-6 (IL-6), tumor necrosis factor-α (TNF-α), monocyte chemotactic protein-1 (MCP-1), and IL-1β, are involved in the pathogenesis of DN [14]. Emerging evidence has indicated that TLR4 was the first identified molecular of TLRs and is positively involved in the development of kidney diseases and DN [15, 16]. TRL4 is up-regulated due to high glucose levels in intrinsic kidney cells which are known to be associated with the pathogenesis of DN [11, 17].

In the present study, we constructed streptozotocin (STZ)-induced DN rat models and high glucose-induced podocytes to investigate the function of SOCS2 in DN and its underlying mechanism.

## RESULTS

### SOCS2 was down-regulated, and TLR4 and NF-κB were up-regulated in renal tissues of DN patients and DN rats

**Figure 1:**
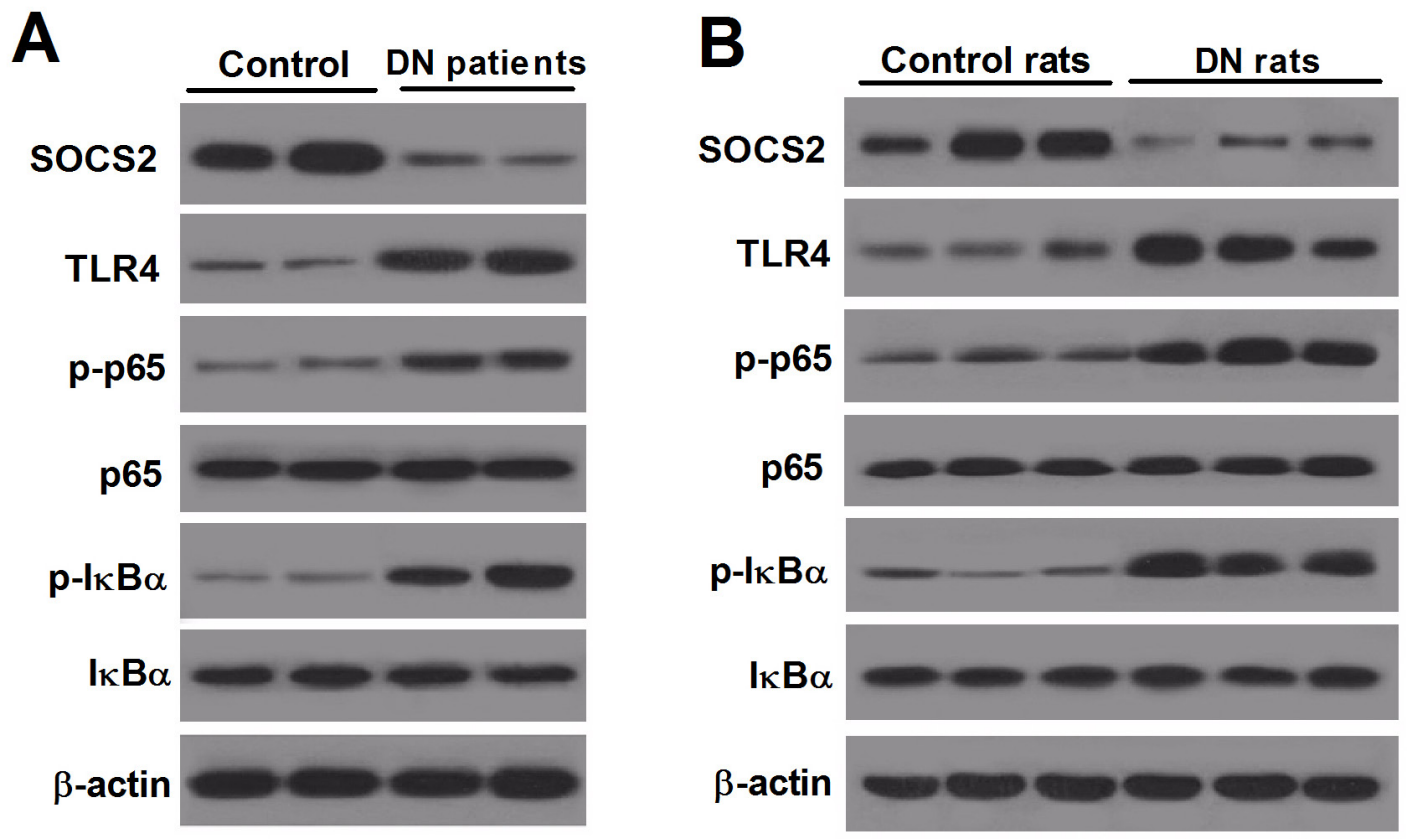
Renal expression of SOCS2, TLR4 and NF-κB in DN patients and DN rats Western blot was performed to determine the levels of renal SOCS2, TLR4, p-p65, p65, p-IκBα and IκBα in DN patients **(A)** and DN rats **(B)**.

Western blot was performed to examine the levels of SOCS2, TLR4 and NF-κB in the renal tissues of DN patients and DN rats. The western blot results showed that the level of SOCS2 was significantly lower and TLR4 was dramatically higher in renal tissues of DN patients (Figure 1A) and DN rats (Figure 1B) than that in control groups. In addition, p65 is one subunit of the NF-κB heterodimer and IκBα regulates the transcriptional activity of NF-κB through the formation of stable IκBα/NF-κB complexes [18]. It is well documented that NF-κB can be activated by the serine phosphorylation of p65 and tyrosine phosphorylation of IκBα [19]. Therefore, determination of NF-κB levels was carried out to detect the phosphorylation of p65 and IκBα. The results indicated that the levels of p-p65 and p-IκBα were markedly increased in the kindey tissue of DN patients (Figure 1A) and DN rats (Figure 1B) in comparison with respective control groups, while the levels of p65 and IκBα had no obvious change. These findings revealed that SOCS2, TLR4 and NF-κB may be involved in the development of DN.

### SOCS2 overexpression alleviated STZ-induced renal injury in DN rats

**Figure 2:**
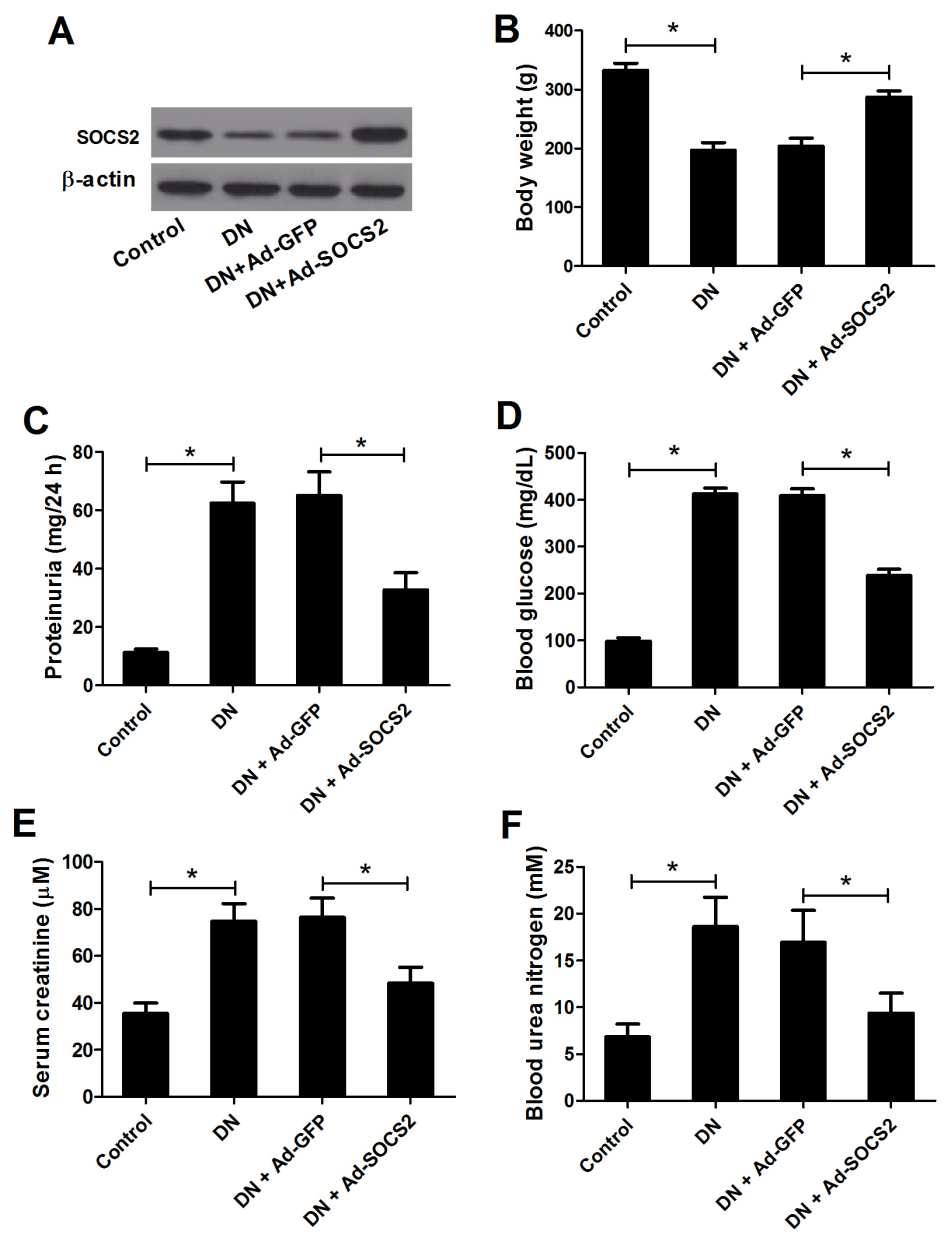
Effect of SOCS2 overexpression on diabetic renal injury **(A)** The expression status of SOCS2 in renal tissues was detected by western blot analysis. The body weight **(B)**, 24-h proteinuria **(C)**, blood glucose level **(D)**, serum creatinine **(E)** and blood urea nitrogen **(F)** were measured in control rats, DN rats or DN rats infected with Ad-GFP or Ad-SOCS2. ^*^P < 0.05.

To investigate the role of SOCS2 in the pathogenesis of DN, recombinant adenovirus vectors with GFP (Ad-GFP) or SOCS2 (Ad-SOCS2) were injected into the kidney tissue of STZ-induced diabetic rats. Four weeks later, the expression levels of SOCS2 protein, determined by western blot analysis, were increased in rats injected with Ad-SOCS2 compared with those in rats injected with Ad-GFP (Figure 2A), suggesting that adenovirus successfully mediated the gene transfer. Also, the body weight, blood glucose level, 24-h proteinuria, serum creatinine and blood urea nitrogen were measured. The body weight of DN rats was markedly decreased compared with control group, while intrarenal injection of adenoviruses carrying SOCS2 attenuated this inhibitory effect (Figure 2B). Additionally, the increase in 24-h proteinuria (Figure 2C), blood glucose level (Figure 2D), serum creatinine (Figure 2E) and blood urea nitrogen (Figure 2F) of DN rats were conspicuously abated by SOCS2 overexpression. These data suggested adenovirus-mediated SOCS2 gene transfer obviously alleviated STZ-induced renal injury in DN rats.

### SOCS2 overexpression inhibited STZ-induced IL-6, IL-1β and MCP-1 generation in DN rats

**Figure 3:**
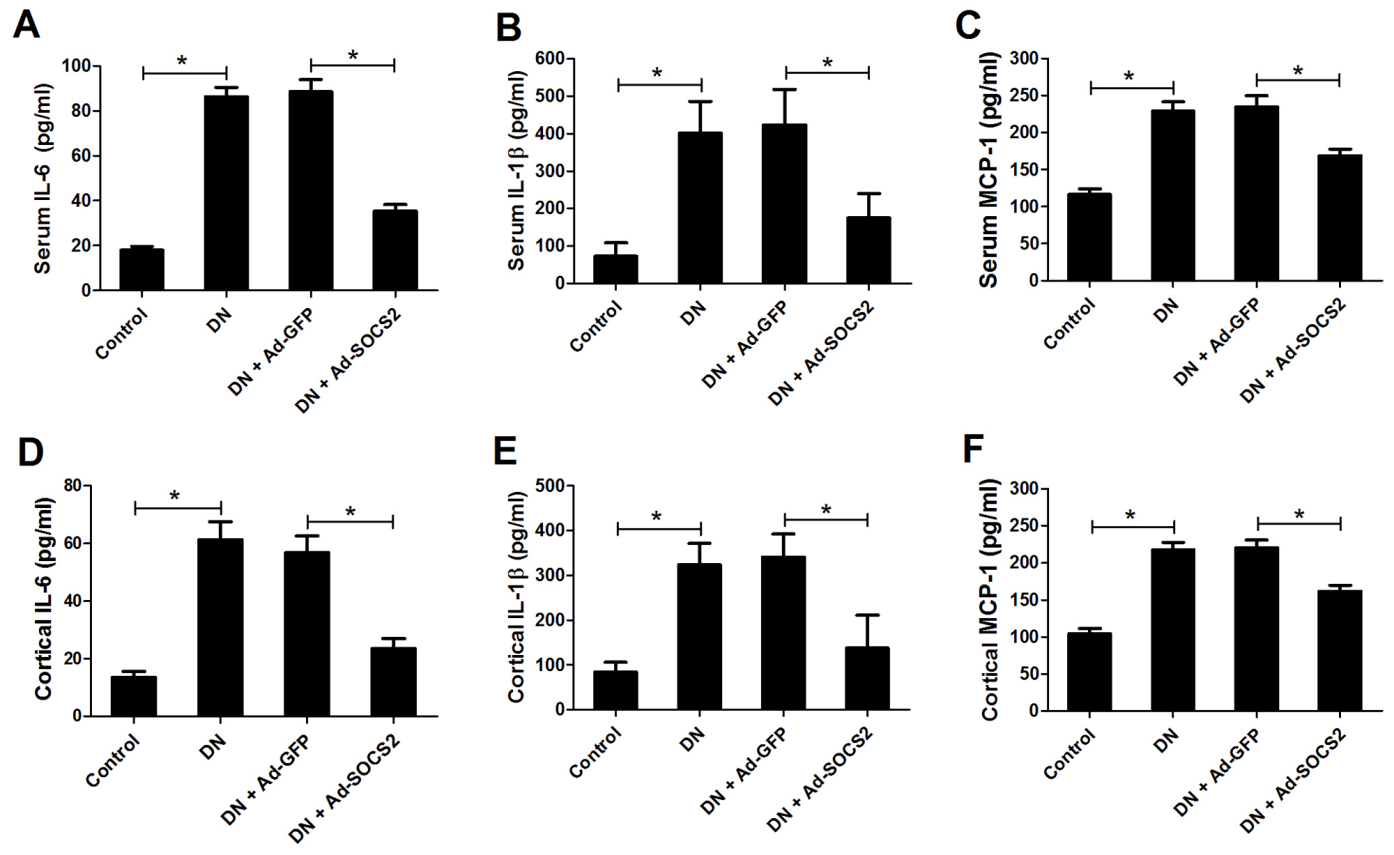
Effect of SOCS2 overexpression on the generation of IL-6, IL-1β and MCP-1 in STZ-induced DN rats The levels of IL-6, IL-1β and MCP-1 in blood **(A, B and C)** and kidney cortex **(D, E and F)** of control rats, DN rats or DN rats infected with Ad-GFP or Ad-SOCS2 were examined by ELISA. ^*^P < 0.05.

Renal inflammation plays a key role in promoting the occurrence and development of DN [20]. To explore whether SOCS2 overexpression could affect STZ-induced inflammatory cytokines production, ELISA was performed to detect the levels of IL-6, IL-1β and MCP-1 in blood and kidney cortex of DN rats. An apparent upregulation was observed in the levels of IL-6, IL-1β and MCP-1 in blood (Figure 3A, 3B and 3C) and kidney cortex (Figure 3D, 3E and 3F) of DN rats, while SOCS2 treatment strikingly inhibited the increase of IL-6, IL-1β and MCP-1 in DN rats. These findings revealed that SOCS2 overexpression distinctly suppressed the production of inflammatory cytokines in DN rats.

### SOCS2 overexpression alleviated STZ-induced renal injury in DN rats

**Figure 4:**
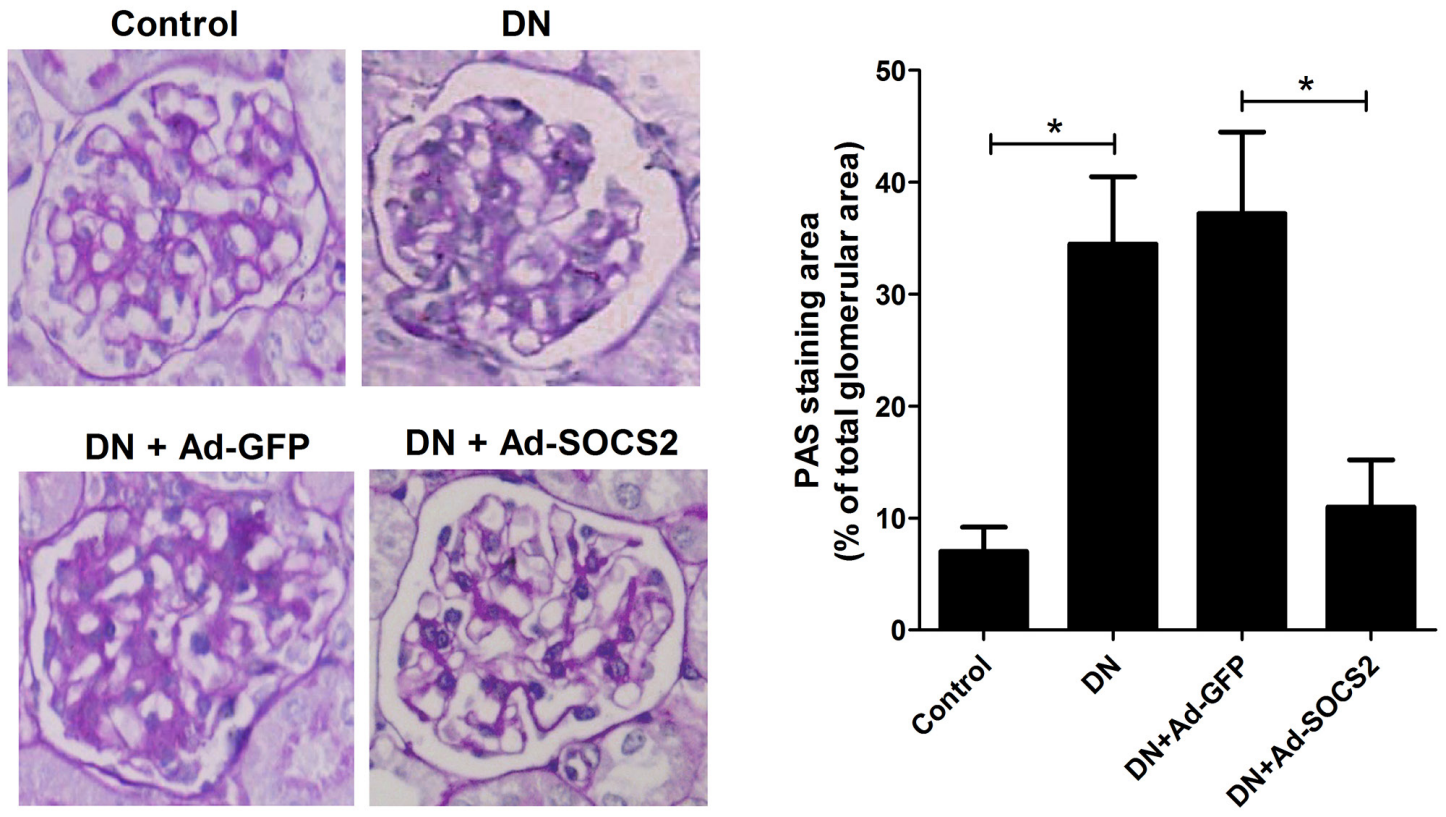
Effect of SOCS2 overexpression on renal injury of DN rats as assessed by PAS staining Representative photomicrographs (400×) of PAS staining of kidney tissues from normal rats, DN rats, DN rats infected with Ad-GFP and Ad-SOCS2 were shown. Extracellular matrix deposition was quantified from PAS staining. ^*^P < 0.05.

PAS staining was carried out to evaluate the renal injury, as shown in Figure 4. Extracellular matrix accumulation was increased in kidney tissues from DN rats compared with that in control rats, indicating a successful DN rat model. Moreover, DN rats infected with Ad-SOCS2 presented a decrease of renal injury as demonstrated by reduced extracellular matrix deposition with respect to that with Ad-GFP infection. These data indicated that SOCS2 upregulation alleviated STZ-induced renal injury in DN rats.

### SOCS2 overexpression suppressed the TLR4/NF-κB signaling pathway in STZ-induced DN rats

**Figure 5:**
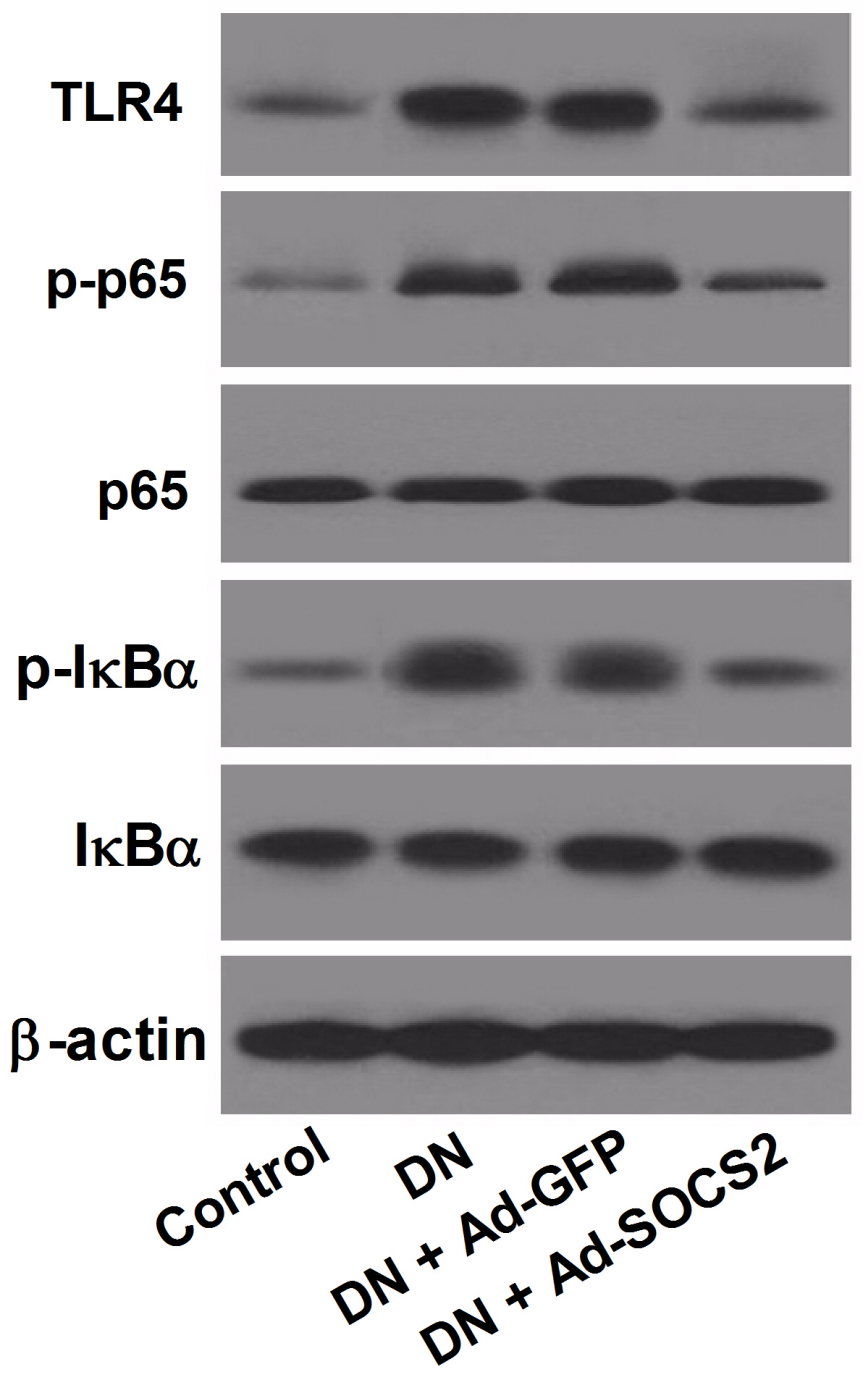
Effect of SOCS2 overexpression on the TLR4/NF-κB signaling pathway in DN rats Western blot was carried out to evaluate the levels of TLR4, p-p65, p65, p-IκBα and IκBα in control rats, DN rats or DN rats infected with Ad-GFP or Ad-SOCS2.

Due to the inverse change of SOCS2 and TLR4/NF-κB in DN patients and DN rats, we next examined the effect of SOCS2 overexpression on the TLR4/NF-κB signaling pathway. The levels of TLR4, p-p65, p65, p-IκBα and IκBα were determined by western blot. The results indicated that the levels of TLR4, p-p65 and p-IκBα was strikingly improved in STZ-induced DN rats compared with that in control rats, whereas SOCS2 overexpression dramatically reversed this effect (Figure 5), indicating that the activation of TLR4/NF-κB signaling pathway in DN rats was markedly inhibited by SOCS2 overexpression.

### SOCS2 overexpression attenuated HG-induced podocyte apoptosis

**Figure 6:**
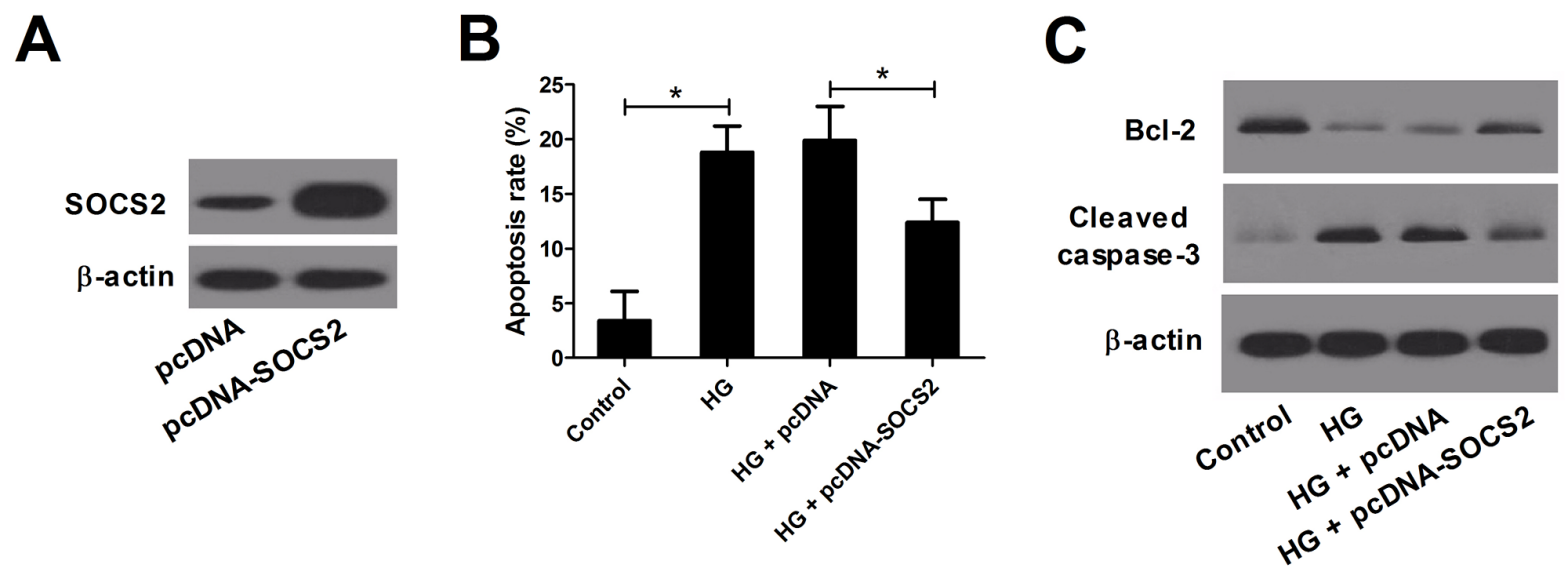
Effect of SOCS2 overexpression on apoptosis in HG-stimulated podocytes Podocytes transfected with pcDNA-SOCS2 or pcDNA were treated with HG for 24 h. **(A)** Western blot analysis was conducted to measure the levels of SOCS2 in podocytes after transfection for 48 h. **(B)** Flow cytometry was performed to assess the apoptosis of treated podocytes. **(C)** Western blot was carried out to evaluate the levels of Bcl-2 and Cleaved caspase-3 in treated podocytes. ^*^P < 0.05.

It has been shown that HG treatment can induce podocyte apoptosis, leading to the onset of DN experimental type 1 and type 2 diabetic models [21]. Therefore, podocyte cells were stimulated with 30 mM D-glucose for 24 h to construct DN cell model. Then, we explored the effect of SOCS2 overexpression on HG-stimulated apoptosis in podocytes. The expression levels of SOCS2 were significantly increased in podocytes transfected with pcDNA-SOCS2 compared with those in podocytes transfected with pcDNA vector (Figure 6A). The flow cytometry analysis exhibited that HG treatment significantly promoted podocyte apoptosis, whereas SOCS2 overexpression dramatically inhibited this effect (Figure 6B). As expected, western blot results uncovered that anti-apoptotic protein Bcl-2 was down-regulated and Cleaved caspase-3 was up-regulated in HG-stimulated podocytes, whereas transfection of pcDNA-SOCS2 markedly overturned these effects (Figure 6C). Taken together, the results revealed that SOCS2 overexpression conspicuously suppressed the apoptosis in HG-induced podocyte.

### SOCS2 overexpression inhibited IL-6, IL-1β and MCP-1 gene expression in HG-stimulated podocytes

**Figure 7:**
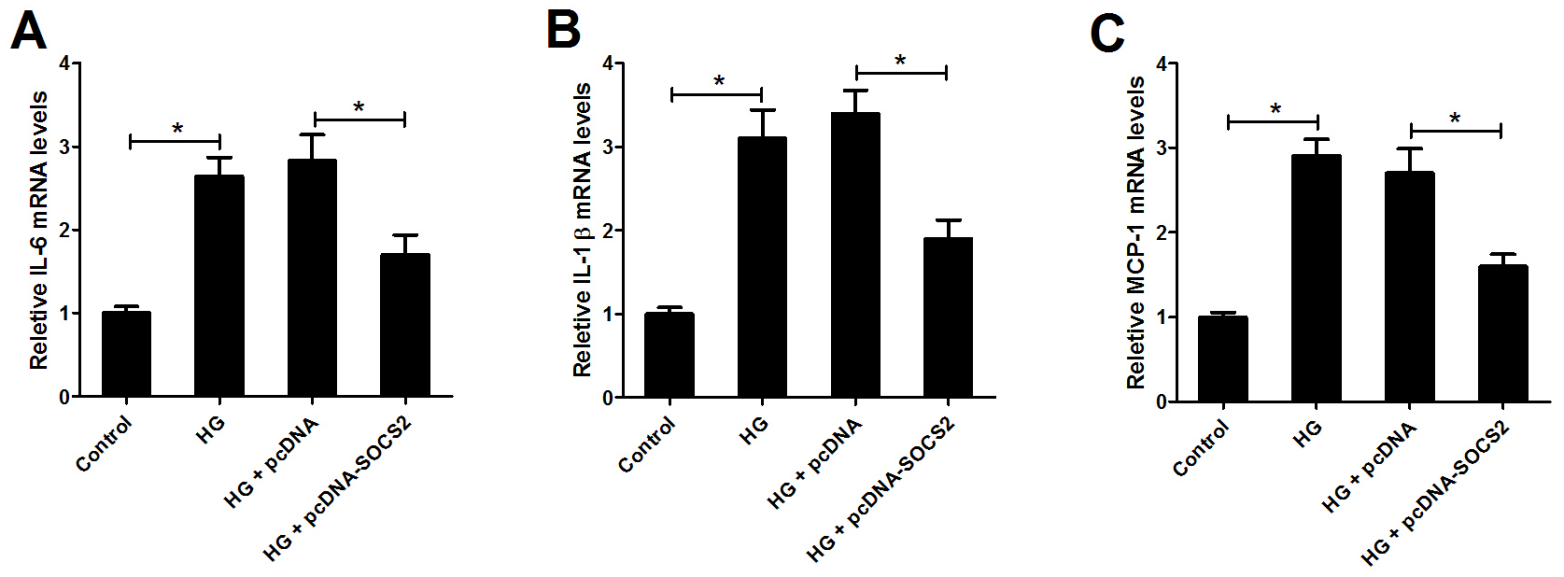
Effect of SOCS2 overexpression on the expression of IL-6, IL-1β and MCP-1 in HG-stimulated podocytes qRT-PCR was used to detect the mRNA expression of IL-6 **(A)**, IL-1β **(B)** and MCP-1 **(C)** in control group, HG group, HG + pcDNA group and HG + pcDNA-SOCS2 group ^*^P < 0.05.

To next examine the effect of SOCS2 on the expression of inflammatory cytokines IL-6, IL-1β and MCP-1 in HG-stimulated podocytes, qRT-PCR was performed. We found that HG treatment markedly increased the expression of IL-6 (Figure 7A), IL-1β (Figure 7B) and MCP-1 (Figure 7C) in podocytes, while SOCS2 upregulation abrogated these effects, indicating that SOCS2 overexpression inhibited the expression of inflammatory cytokines in HG-stimulated podocytes.

### SOCS2 overexpression showed a stronger inhibitory effect on the TLR4/NF-κB pathway than the JAK/STAT pathway in HG-stimulated podocytes

**Figure 8:**
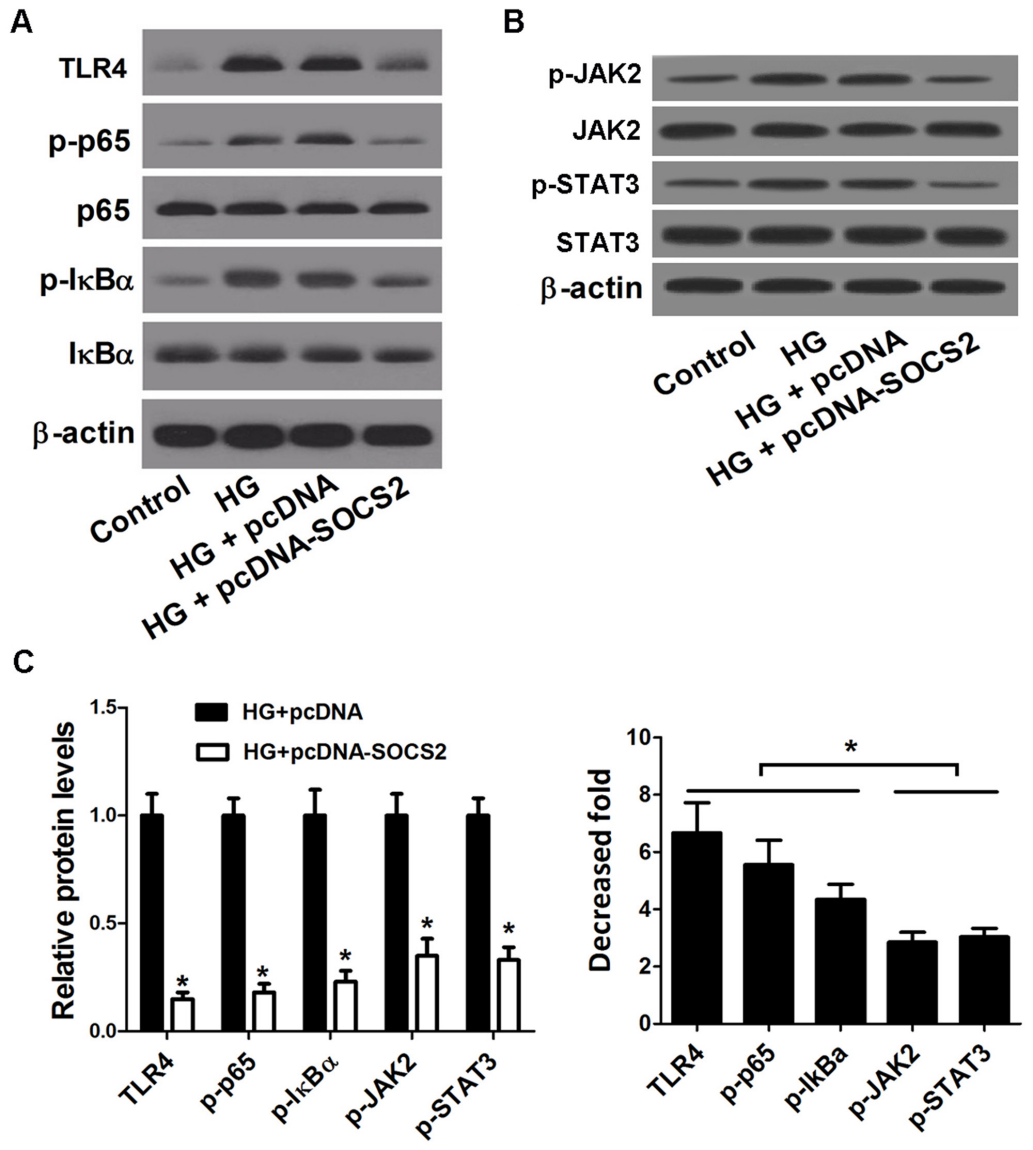
Effect of SOCS2 overexpression on the TLR4/NF-κB pathway and the JAK/STAT pathway in HG-stimulated podocytes **(A)** Western blot was carried out to determine the expression of TLR4, p-p65, p65, p-IκBα and IκBα in control podocytes, HG-stimulated podocytes or HG-stimulated podocytes transfected with pcDNA-SOCS2 or pcDNA. **(B)** Western blot was conducted to analyze the protein levels of p-JAK2, JAK2, p-STAT3, and STAT3 in control podocytes, HG-stimulated podocytes or HG-stimulated podocytes transfected with pcDNA-SOCS2 or pcDNA. **(C)** The decrease fold of the protein levels of TLR4, p-p65, p-IκBα, p-JAK2, and p-STAT3 in HG-stimulated podocytes or HG-stimulated podocytes transfected with pcDNA-SOCS2 or pcDNA. ^*^P < 0.05.

The effect of SOCS2 overexpression on the TLR4/NF-κB pathway in HG-stimulated podocytes was further analyzed. Western blot results showed that the levels of TLR4, p-p65 and p-IκBα were conspicuously higher in HG-stimulated podocytes than that in control group. However, SOCS2 transfection led to a significant decrease of TLR4, p-p65 and p-IκBα in HG-stimulated podocytes (Figure 8A), suggesting that SOCS2 overexpression repressed the activation of TLR4/NF-κB pathway in HG-stimulated podocytes. Additionally, a previous study demonstrated that overexpression of SOCS2 suppressed the activation of the JAK/STAT signaling pathways mediated by DN. As expected, ectopic expression of SOCS2 significantly suppressed the phosphorylation of JAK2 and STAT3 in HG-stimulated podocytes (Figure 8B). We further compared the effect of SOCS2 on the JAK/STAT pathway with the TLR4/NF-κB pathway in HG-stimulated podocytes. As presented in Figure 8C, overexpression of SOCS2 both markedly inhibited the TLR4/NF-κB pathway and the JAK/STAT pathway; however, the decrease fold of the related proteins in the TLR4/NF-κB pathway was significantly higher than the JAK/STAT pathway, indicating TLR4/NF-κB pathway, are the major downstream mediator in SOCS2 upregulation-mediated protection of DN.

### SOCS2 overexpression suppressed apoptosis and inflammatory cytokines expressions mainly by inhibiting the TLR4/NF-κB pathway in HG-stimulated podocytes

**Figure 9:**
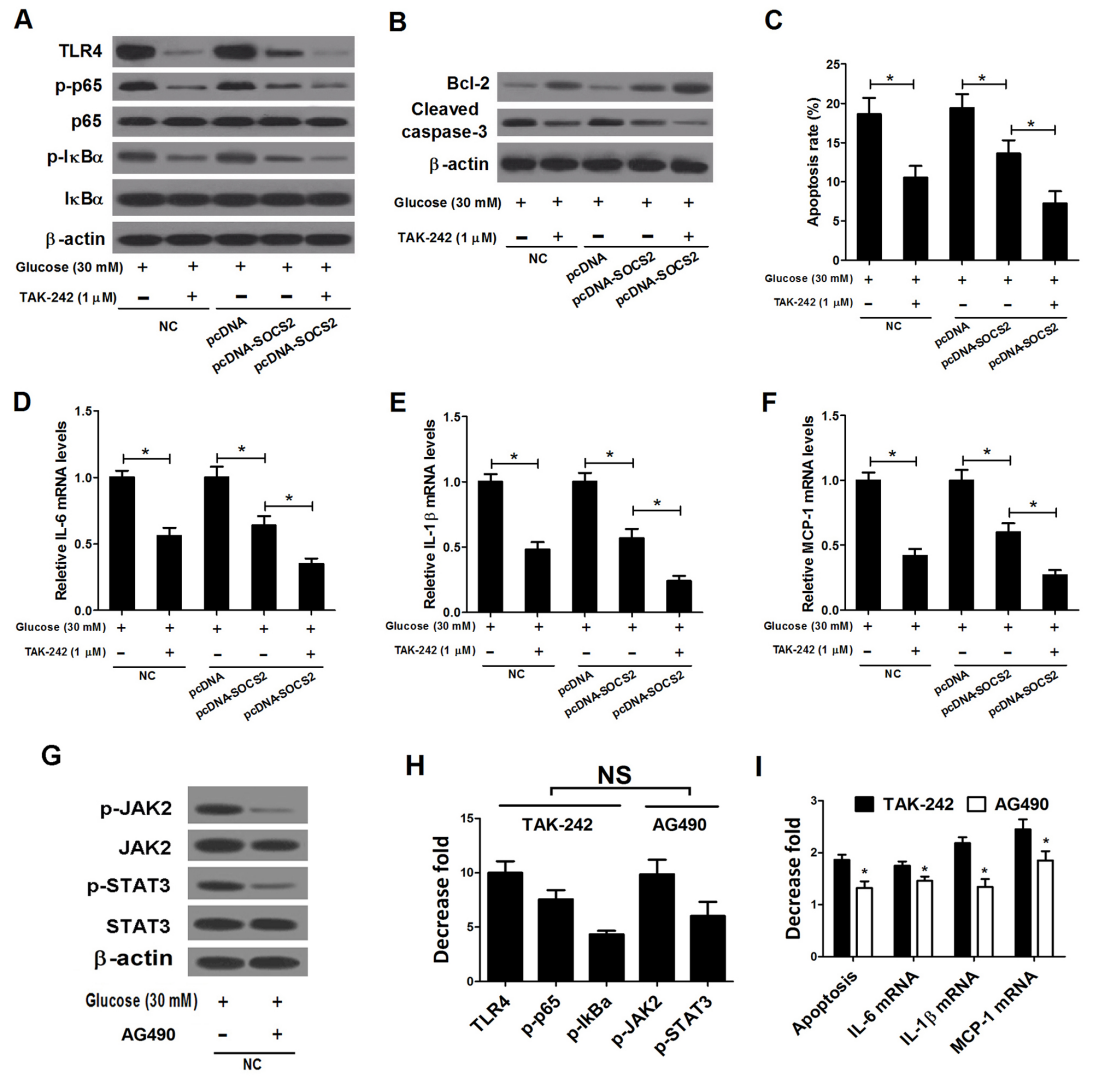
Effects of TAK-242 and AG490 on apoptosis and inflammatory cytokines expressions in HG-stimulated podocytes transfected with pcDNA-SOCS2 Non-transfected or transfected (pcDNA or pcDNA-SOCS2) podocytes were pretreated with or without 1 μM TAK-242 for 2 h, and then stimulated with 30 mM glucose for 24 h. **(A)** Non-transfected or transfected (pcDNA or pcDNA-SOCS2) podocytes were pretreated with or without 1 μM TAK-242 for 2 h, and then stimulated with 30 mM glucose for 24 h. Western blot was then performed to detect the levels of TLR4, p-p65, p65, p-IκBα and IκBα in treated podocytes. **(B)** Non-transfected or transfected (pcDNA or pcDNA-SOCS2) podocytes were pretreated with or without 1 μM TAK-242 for 2 h, and then stimulated with 30 mM glucose for 24 h. The levels of Bcl-2 and Cleaved caspase-3 in treated podocytes were examined by western blot. **(C)** Non-transfected or transfected (pcDNA or pcDNA-SOCS2) podocytes were pretreated with or without 1 μM TAK-242 for 2 h, and then stimulated with 30 mM glucose for 24 h. Apoptosis of treated podocytes was assessed by flow cytometry. The mRNA expressions of IL-6 **(D)**, IL-1β **(E)** and MCP-1 **(F)** in treated podocytes were determined by qRT-PCR. **(G)** Podocytes were pretreated with or without 20 μM AG490 for 30 min, and then stimulated with 30 mM glucose for 24 h. The effect of AG490 on the JAK/STAT pathway in treated podocytes was explored by western blot. **(H)** The decrease fold of the protein levels of TLR4, p-p65, p-IκBα in TAK-242-treated podocytes, and the protein levels of p-JAK2, and p-STAT3 in AG490-treated podocytes. **(I)** The decrease fold of apoptosis and mRNA expressions of inflammatory cytokines (IL-6, IL-1β and MCP-1) in TAK-242 or AG490-treated podocytes. ^*^P < 0.05.

TAK-242, a molecularly-targeted clinical TLR4 antagonist, was applied to inhibit the TLR4/NF-κB pathway to investigate the mechanism of SOCS2 function in HG-stimulated podocytes. The western blot results showed that either TAK-242 or SOCS2 overexpression significantly reduced the levels of TLR4, p-p65 and p-IκBα in HG-stimulated podocytes, and TAK-242 enhanced the pcDNA-SOCS2-induced inhibitory effect on TLR4, p-p65 and p-IκBα expression (Figure 9A). Both TAK-242 and SOCS2 overexpression resulted in a significant promotion of Bcl-2 level and an obvious inhibition of Cleaved caspase-3 level in HG-stimulated podocytes, moreover, combination of TAK-242 treatment and SOCS2 overexpression led to a more prominent effect (Figure 9B). Similarly, TAK-242 or SOCS2 upregulation significantly repressed apoptotic rates of HG-stimulated podocytes, while simultaneous TAK-242 treatment and pcDNA-SOCS2 transfection strengthened this effect (Figure 9C). Additionally, a significant reduction of the expressions of inflammatory cytokines IL-6 (Figure 9D), IL-1β (Figure 9E) and MCP-1 (Figure 9F) was observed due to TAK-242 treatment or pcDNA-SOCS2 transfection in HG-stimulated podocytes, and TAK-242 reinforced the inhibitory influence of SOCS2 overexpression on these inflammatory cytokines. AG490, a synthetic inhibitor to the JAK/STAT pathway, was used to inhibit the JAK/STAT pathway and the results indicated that the JAK/STAT pathway was dramatically repressed by AG490 (Figure 9G). In addition, it is proved that TAK-242 and AG490 exhibited the same inhibitory effect on the TLR4/NF-κB pathway and the JAK/STAT pathway, respectively (Figure 9H). However, TAK-242 showed a higher decrease fold of apoptosis and the mRNA expressions of inflammatory cytokines (IL-6, IL-1β and MCP-1) than AG490 (Figure 9I). Taken together, these data implicated that the inhibitory influence of SOCS2 overexpression on apoptosis and inflammatory cytokines in HG-stimulated podocytes was mediated mainly by inactivation of TLR4/NF-κB pathway.

### PDTC reinforced the negative effect of SOCS2 upregulation on apoptosis and inflammatory cytokines expressions in HG-stimulated podocytes

**Figure 10:**
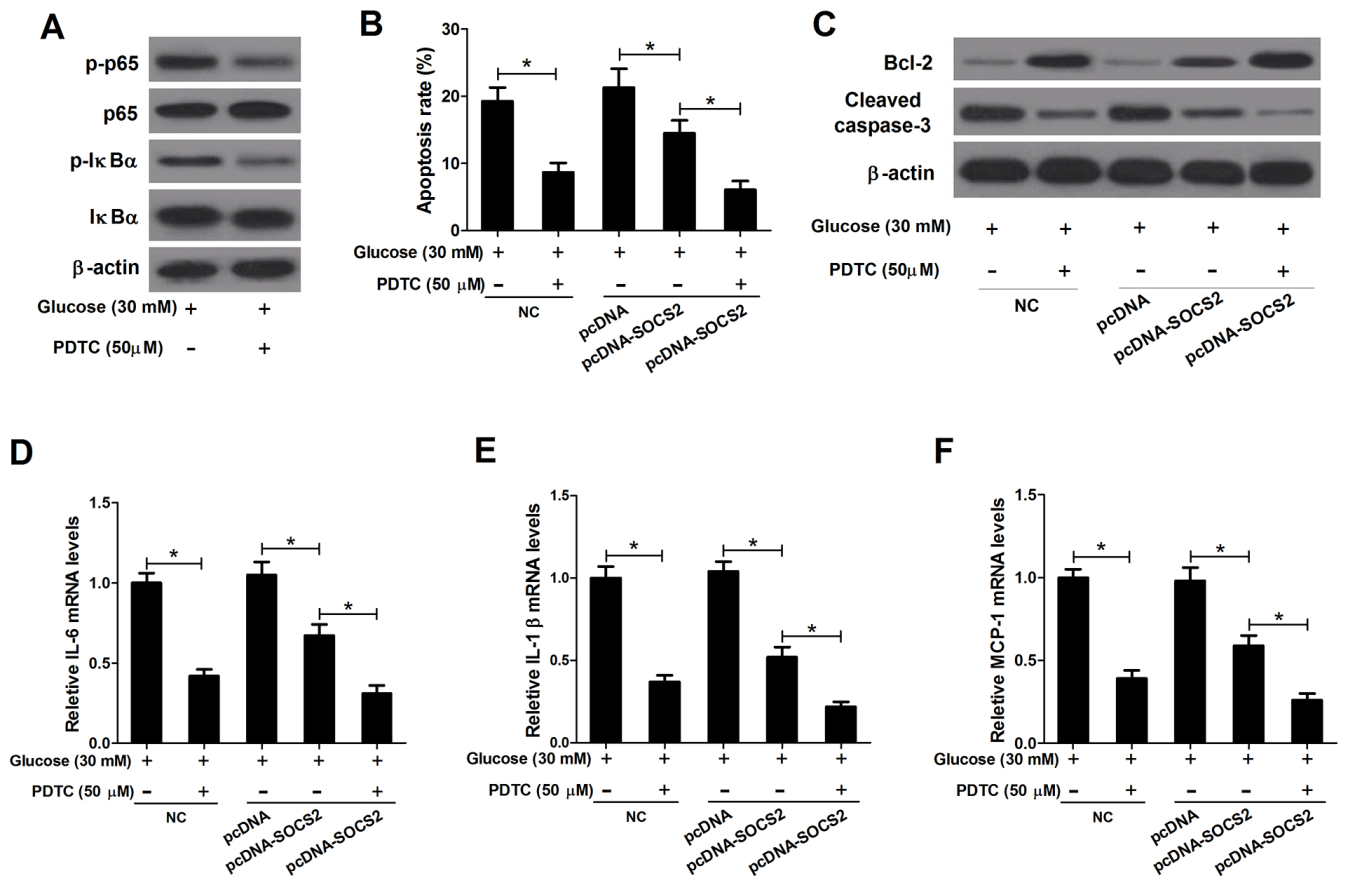
Effect of PDTC on apoptosis and inflammatory cytokines expressions in HG-stimulated podocytes transfected with pcDNA-SOCS2 Non-transfected or transfected (pcDNA or pcDNA-SOCS2) podocytes were preconditioned with or without 50 μM PDTC for 2 h, followed by exposure to 30 mM glucose for 24 h. **(A)** The levels of p65, p-p65, IκBα, and p-IκBα were detected by western blot analysis. **(B)** Apoptosis of treated podocytes was examined by flow cytometry. **(C)** The levels of Bcl-2 and Cleaved caspase-3 in treated podocytes were determined by western blot. The mRNA expressions of IL-6 **(D)**, IL-1β **(E)** and MCP-1 **(F)** in treated podocytes were determined by qRT-PCR. ^*^P < 0.05.

PDTC, an inhibitor of NF-κB, was used to further verify the mechanism of SOCS2 action in HG-stimulated podocytes. As shown in Figure 10A, PDTC decreased the expression levels of p-p65 and p-IκBα in the presence of HG treatment. Either PDTC treatment or pcDNA-SOCS2 transfection exerted a significant inhibitory role in apoptosis in HG-stimulated podocytes, and combination treatment led to a higher apoptosis rate (Figure 10B). Besides, the level of Bcl-2 was improved and Cleaved caspase-3 expression was reduced due to PDTC treatment or SOCS2 overexpression, while simultaneous PDTC treatment and pcDNA-SOCS2 transfection enhanced these effects (Figure 10C). Furthermore, either PDTC treatment or SOCS2 upregulation led to a significant decrease of IL-6 (Figure 10D), IL-1β (Figure 10E) and MCP-1 (Figure 10F) expressions in HG-stimulated podocytes, and PDTC augmented pcDNA-SOCS2-induced increase of inflammatory cytokines expression. These results further confirmed that SOCS2 overexpression induced suppression of apoptosis and inflammatory cytokines expressions by inhibiting the TLR4/NF-κB pathway in HG-stimulated podocytes.

## DISCUSSION

It has been implied that glucose metabolic disturbance, oxidative stress, inflammatory reaction and abnormal hemodynamics play a crucial role in the progression and development of DN [6, 22]. It is meaningful to better understand the pathogenesis of DN for developing effective therapeutic strategies against DN.

It has been shown that SOCS2 acts as an important regulator of inflammatory responses and is associated with the pathogenesis of DN [23, 24]. Zhou *et al.* reported that SOCS2 attenuated STZ-induced renal lesions including renal/glomerular hypertrophy, glomerular hyperfiltration, aberrant inflammation and fibrosis and reduced the levels of proinflammatory proteins (TGF-β, collagen IV and fibronectin) in DN [25]. Bao *et al.* found that SOCS2 overexpression decreased the increased expressions of inflammatory cytokines (MCP-1, TNF-α and IL-6) and fibrosis related protein in STZ-induced DN rats [9]. Consistently, our study showed that SOCS2 overexpression significantly increased the body weight and dramatically decreased the 24-h proteinuria which is a significant clinical hallmark of early stage DN, blood glucose level, serum creatinine and blood urea nitrogen in DN rats, alleviating diabetic renal injury. PAS staining demonstrated that SOCS2 overexpression led to a decrease of glomerulosclerosis and renal interstitial fibrosis. Meanwhile, SOCS2 overexpression also inhibited the production of inflammatory cytokines (IL-6, IL-1β and MCP-1) in DN rats and HG-stimulated podocytes. Besides, SOCS2 upregulation significantly attenuated HG-induced podocyte apoptosis. These results suggested that SOCS2 overexpression could alleviate the development of DN.

TLR4 signaling pathway can activate downstream inflammation response signaling pathways such as the NF-κB pathway and result in the release of inflammatory cytokines and chemokines causing inflammation, contributing to the pathogenesis of inflammation-associated renal injury and kidney disease [26, 27]. Ma *et al.* exhibited that TLR4 activation promoted inflammation, NF-κB activation, podocyte and tubular epithelial cell injury, and interstitial fibrosis in DN [12]. Lin *et al.* noticed that TLR4 was up-regulated in renal tubules of human kidneys with diabetic nephropathy and high glucose induced TLR4 expression via protein kinase C activation, resulting in up-regulation of IL-6 and chemokine (C-C motif) ligand 2 (CCL-2) by IκB/NF-κB activation in human tubular epithelial cells, contributing to tubulointerstitial inflammation in DN [12]. In our study, we found that SOCS2 overexpression markedly inhibited the activation of the TLR4/NF-κB signaling pathway in DN rats and HG-stimulated podocytes. Furthermore, the TLR4 antagonist (TAK-242) and NF-κB inhibitor (PDTC) were used to inhibit the TLR4/NF-κB signaling pathway to further investigate the mechanism of SOCS2 in the pathogenesis of DN. TAK-242 and PDTC both conspicuously suppressed apoptosis and inflammatory cytokines production in HG-stimulated podocytes; moreover, either TAK-242 or PDTC strengthened the inhibitory influence of SOCS2 overexpression on apoptosis and inflammatory cytokines, indicating that SOCS2 overexpression repressed apoptosis and inflammatory cytokines production by inhibiting the TLR4/NF-κB signaling pathway in HG-stimulated podocytes. Additionally, upregulation of SOCS2 has been demonstrated to suppress JAK/STAT and EPK signaling pathways induced by STZ in rat kidneys [9, 24], consistent with our present results. Our study demonstrated that SOCS2 overexpression showed a stronger inhibitory effect on the TLR4/NF-κB pathway than the JAK/STAT pathway in HG-stimulated podocytes. Furthermore, TAK-242 showed a higher decrease fold of apoptosis and the mRNA expressions of inflammatory cytokines (IL-6, IL-1β and MCP-1) than AG490, suggesting that SOCS2 overexpression suppressed apoptosis and inflammatory cytokines expressions mainly by inhibiting the TLR4/NF-κB pathway in HG-stimulated podocytes.

In conclusion, we demonstrated that SOCS2 adenovirus injection could relieve STZ-induced diabetic renal injury and decreased the generation of inflammatory cytokines in DN rats. SOCS2 overexpression attenuated the apoptosis and inhibited inflammatory cytokines secretions in HG-stimulated podocytes. We further illustrated that SOCS2 overexpression suppressed the apoptosis and inflammatory cytokines expression mainly by inhibiting the TLR4/NF-κB signaling pathway in HG-stimulated podocytes. This study suggested that inactivation of TLR4/NF-κB signaling pathway via SOCS2 may be an effective strategy for DN patients.

## MATERIALS AND METHODS

### Patients and tissue samples

A total of 22 renal biopsies from advanced DN patients and 9 healthy renal biopsies were recruited from Huaihe Hospital of Henan University and were snap-frozen in liquid nitrogen. All DN patients were histopathologically diagnosed with diffuse or nodular diabetic glomerulosclerosis and no other renal pathology. Written informed consent was obtained from all of the participants before specimen collection and the study was approved by the Ethics Committee of Huaihe Hospital of Henan University which was in compliance with the Helsinki declaration.

### Animal model and grouping

Male Sprague-Dawley (SD) rats (8 weeks old, weighting 180-220 g) were purchased from Chinese Academy of Medical Sciences (Beijing, China). The rats were housed in a conventional environment with a regular light/dark cycle of 12 h, as well as a standard diet and tap water. All animal experiments were approved by the Animal Care Committee of Huaihe Hospital of Henan University (China). The rat models of DN were established by an administration of intraperitoneal injection of STZ at 60 mg/kg (dissolved in 0.01 M citrate buffer, pH 4.5; Sigma, St. Louis, MO, USA) followed by a high-glucose-fat diet (formula: 69% standard rat feed, 1% cholesterol, 0.5% sodium cholate, 15% lard and 10% carbohydrate). At 72 h after administration, their blood glucose levels were measured. If random blood glucose levels ≥ 16.7 mmoL/L, the diabetic model rats were considered to be successfully prepared. Rats treated with equivalent amounts of buffer injection were used as controls.

At 8 weeks after STZ injection, DN-model rats were anesthetized by 10% of chloral hydrate and the left renal vein was exposed via midline incision. Recombinant adenovirus vectors expressing SOCS2 gene (Ad-SOCS2; Hanheng Bioscience Incorporation, Shanghai, China) or carrying green fluorescent protein gene (Ad-GFP; Hanheng Bioscience Incorporation) as negative control adenoviruses were infused into the renal vein. Then the rats were randomly assigned into 3 groups (n=6 per group): (1) DN; (2) DN + Ad-GFP, rats receiving an injection of 0.6 mL recombinant adenovirus vectors carrying green fluorescent protein gene (Ad-GFP); (3) DN + Ad-SOCS2, rats receiving an injection of 0.6 mL recombinant adenovirus vectors expressing SOCS2 gene. After 4 weeks, the blood and 24-h urine samples of rats were collected. Thereafter, rats were euthanatized and the kidney tissues were removed and frozen at −80°C for further analysis.

### Cell culture, model and transfection

The conditionally immortalized podocyte cells were cultured as previously described [28]. In brief, cells were maintained in RPMI 1640 medium (Gibco, Grand Island, NY, USA) supplemented with 100 U/ml recombinant-IFNγ (Invitrogen, Grand Island, NY) and 10% fetal bovine serum (FBS, Invitrogen) at the permissive temperature 33°C. To induce DN cell model, podocyte cells were stimulated with 30 mM D-glucose (high glucose, HG) for 24 h. Transient transfection with 4 μg pcDNA-SOCS2 or pcDNA empty vector (GenePharma, Shanghai, China) were carried out in podocyte cultured in six-well plates by Lipofectamine^®^ 2000 (Invitrogen). 1 μM TAK-242 or 50 μM PDTC was used to inhibit the TLR4/NF-κB pathway in podocyte. 20 μM AG490 was used to suppress the Janus kinase (JAK)/signal transducers and activators of transcription (STAT) pathway in podocyte.

### Quantitative real-time PCR (qRT-PCR)

Total RNA from podocyte cells treated with HG alone or in combination with pcDNA-SOCS2 or pcDNA was extracted by Trizol reagent (Invitrogen). The quality and purity of extracted RNA were evaluated by a NanoDrop Spectrophotometer (Thermo Scientific, Hudson, NH, USA) at 260 nm and 280 nm. 2 μg of total RNA was reverse transcribed by High-Capacity cDNA Reverse Transcription Kit (Thermo Fisher Scientific, Waltham, MA, USA). Real-time PCR was carried out using FastStart SYBR Green Master mix (Roche Applied Science, Mannheim, Germany) according to the manufacturer’s instructions on a ViiA™ 7 Real-Time PCR System (Applied Biosystems, Foster City, CA, USA) with the following protocol: 10 min at 95°C, followed by 45 cycles of 95°C for 15 s, 60°C for 25 s and 72°C for 25 s. Relative gene expression were calculated by the 2^−ΔΔCt^ method and normalized to the internal control GAPDH expression.

### Western blot

Rat renal biopsies samples and cultured podocyte cells were placed on ice to lyse in cell lysis buffer (Beyotime, Haimen, China) and centrifuged at 12000 rpm for 10 min at 4°C. The total protein in the cell supernatant of each sample was detected using the Bradford method (Pierce, Rockford, IL, USA). An equal amount of protein (25 μg) of each sample was separated by 10% sodium dodecyl sulfate-polyacrylamide gel electrophoresis (SDS-PAGE) and transferred to polyvinylidene fluoride membranes (PVDF; Bio-Rad, Hercules, CA, USA). After blocking with 2.5% skim milk for 1 h at 37°C, the members were then incubated with primary antibodies against SOCS2, TLR4, p-p65, p65, p-IκBα, IκBα, Bcl-2, Cleased caspase-3 and β-actin (1:1000; Abcam, Cambridge, MA, USA) at 4°C overnight. The members were then incubated with HRP-conjugated anti-rabbit antibody (1:1000; Santa Cruz, Waltham, MA, USA) for 2 h at room temperature and visualized by using the enhanced chemiluminescence (ECL) system (Pierce).

### Enzyme-linked immunosorbent assay (ELISA)

Determination of inflammatory cytokines IL-6, IL-1β and MCP-1 levels in blood and kidney cortex of STZ-induced DN rats were performed by a specific ELISA kit (Wuhan Boster Biological Technology, Ltd., Wuhan, China).

### Analysis of body weight, proteinuria, blood glucose, serum creatinine and blood urea nitrogen

The body weight of each diabetic model rats was measured 4 weeks after administration of Ad-GFP or Ad-SOCS2 infection. The collected urine specimens from all rats were centrifuged at 3000 rpm for 10 minutes at 4°C. The supernatant was measured for proteinuria concentration by ELISA. The right jugular artery was catheterized and the blood samples collected from all rats was centrifuged at 3000 rpm for 10 minutes at 4°C. The concentrations of blood glucose were examined by a diagnostic kit (Applied Biosystems). The concentration of serum creatinine was analyzed by an automatic biochemistry analyzer (Hitachi Model 7600, Shibuya, Tokyo, Japan). The concentration of blood urea nitrogen was finally tested by a kinetic reagent (Diagnostic Chemicals Limited, Oxford, Connecticut, USA).

### Flow cytometry analysis

Apoptosis of cultured human podocyte cells in different groups was examined using an Annexin V/PI apoptosis detection kit according to manufacturer’s protocol (Nanjing KeyGEN Biotech, Nanjin, China). A FACScan flow cytometer with Cell Quest software (BD Biosciences, San Jose, CA, USA) was used to count apoptotic cells that were annexin V-positive and propidium iodide-negative.

### PAS staining

Renal sections from diabetic model rats were detected by periodic acid-Schiff (PAS) staining to depict renal pathological changes. The PAS positive areas are indicative of early necrotic damage. Briefly, tissues were deparafinized, rehydrated, oxidized in 0.5% periodic acid (Richard-Allan Scientific, Kalamazoo, MI, USA) for 5 min at 60°C and washed with distilled water for 5 min. The tissues were then stained with Schiff’s reagent (Richard-Allan Scientific) for 10 min and washed again. Thereafter, tissues were counterstained with Harris’s haematoxylin (Sigma) for 1 min and immersed in Bluing reagent for 10 s. In each section, ten fields (200× magnification) were randomly selected and viewed using a microscope (Olympus BX51, Japan) The quantitative analysis on 10 glomeruli per kidney section was performed using an image analysis system (Image Pro Plus 6.0, Media Cybernetics, Silver Spring, MD, USA).

### Statistical analysis

SPSS 17.0 software (SPSS Inc., Chicago, IL, USA) was used to perform the statistical analyses. The results were presented as mean ± standard deviation (SD). Multiple group comparisons were determined by one-way ANOVA. Statistical significance was accepted at *P* < 0.05.

## Abbreviations

SOCS2: Suppressor of cytokine signaling 2
DN: Diabetic Nephropathy
TLR4: Toll-like receptors 4
STZ: streptozotocin
NF-κB: nuclear factor kappa B
HG: high glucose
ESRD: end-stage renal disease
JAK: Janus kinase
STAT: signal transducers and activators of transcription
TLRs: Toll-like receptors
PAMPs: pathogen associated molecular patterns
HSPs: heat-shock proteins
NF-κB: factor kappa B
IL-6: interleukin-6
TNF-α: factor-α
MCP-1: monocyte chemotactic protein-1
SD: Sprague-Dawley
